# Immunoregulatory roles of post-translational modifications in colorectal cancer: mechanisms and therapeutic implications

**DOI:** 10.1007/s00018-025-05992-3

**Published:** 2025-12-08

**Authors:** Xinyue Liang, Jinhong Yao, Wenbo Jiao, Xiaolin Li, Bo Yang, Hongqiong Fan

**Affiliations:** 1 Departments of Hematology, The First Hospital of Jilin University, Changchun, Jilin 130021 China; 2 Department of Cardiothoracic Surgery, The First Hospital of Jilin University, Changchun, Jilin 130021 China; 3 Department of Clinical Laboratory, the Affiliated Hospital of Changchun University of Chinese Medicine, No. 1478, Gongnong Avenue, Changchun, Jilin 130021 China; 4 Department of Infection Control, The Affiliated Hospital of Changchun University of Chinese Medicine, No.2 Shenzhen Street, Changchun, Jilin 130000 China; 5 Department of Thoracic Surgery, The First Hospital of Jilin University, Changchun, Jilin 130021 China

**Keywords:** Phosphorylation, Ubiquitination, T cell exhaustion, Macrophage polarization, Immune checkpoint blockade

## Abstract

Colorectal cancer (CRC) remains a leading cause of cancer morbidity and mortality worldwide, with tumor immune evasion posing a major challenge to effective immunotherapy. Post-translational modifications (PTMs), including phosphorylation, ubiquitination, acetylation, methylation, and glycosylation, are critical regulators of protein function and stability, profoundly influencing tumor immunogenicity and the tumor immune microenvironment. This review comprehensively examines how PTMs modulate key immune processes in CRC, such as antigen presentation, immune cell infiltration, and immune checkpoint regulation. We discuss PTM-mediated mechanisms that shape T cell exhaustion, macrophage polarization, and immunosuppressive cytokine networks within the tumor microenvironment. Moreover, we highlight the impact of PTMs on therapeutic response and resistance to immune checkpoint blockade and adoptive cell therapies. Emphasis is placed on emerging PTM-targeted strategies to enhance antitumor immunity and overcome immunotherapy resistance. Finally, we explore advances in multi-omics technologies and proteomic profiling that promise to accelerate the identification of PTM biomarkers and novel therapeutic targets. By integrating mechanistic insights with translational perspectives, this review aims to provide a foundation for leveraging PTMs to optimize immunotherapeutic approaches in colorectal cancer.

## Introduction

Colorectal cancer (CRC) ranks among the most prevalent malignancies worldwide and remains a leading cause of cancer-related mortality [1]. According to the Global Cancer Statistics 2020, CRC accounts for nearly 10% of all new cancer cases and deaths globally, with an increasing incidence in both developed and developing countries([2]– [3]). Despite advances in screening and early detection, a significant proportion of patients present with advanced or metastatic disease at diagnosis, which is associated with poor prognosis. Standard treatments including surgery, chemotherapy, and targeted therapies have improved survival outcomes; however, the 5-year survival rate for metastatic CRC remains below 15% [4]. The heterogeneity of CRC, driven by diverse genetic, epigenetic, and microenvironmental factors, poses substantial challenges for effective management([5]– [6]).

Over the past decade, immunotherapy, particularly immune checkpoint blockade (ICB), has revolutionized cancer treatment([7]– [8]). In CRC, however, clinical benefits of immunotherapy are largely restricted to a subset of patients exhibiting microsatellite instability-high (MSI-H) or mismatch repair-deficient (dMMR) tumors, which represent approximately 15% of all cases([9]– [10]). These tumors are characterized by high mutational burden and robust immune infiltration, rendering them more responsive to checkpoint inhibitors targeting PD-1/PD-L1 or CTLA-4. By contrast, microsatellite-stable (MSS) CRC, the immunologically “cold” phenotype representing the remaining ~ 85%, exhibits limited responsiveness to current immunotherapies, largely due to a profoundly immunosuppressive tumor microenvironment([11]– [12]). Immune evasion in CRC involves a complex interplay of impaired antigen presentation, immune checkpoint overexpression, T cell dysfunction, and infiltration by immunosuppressive myeloid cells. Elucidating the molecular circuits governing these processes is essential for expanding the scope of immunotherapy beyond the MSI-H/dMMR subgroup.

Post-translational modifications (PTMs) refer to the covalent and enzymatic modifications of proteins following translation, which dynamically regulate protein activity, stability, localization, and interactions. PTMs encompass a diverse array of chemical modifications, including phosphorylation, ubiquitination, acetylation, methylation, glycosylation, and emerging types such as SUMOylation and lactylation. In recent years, PTMs have emerged as pivotal modulators of cancer biology, not only influenced tumor cell intrinsic pathways but also profoundly shaping the tumor immune landscape. For instance, PTMs regulate the expression and function of immune checkpoints, antigen processing and presentation machinery, and signaling cascades that govern immune cell activation and exhaustion [13]. Moreover, PTMs orchestrate the phenotype and function of stromal and immune cells within the tumor microenvironment (TME), modulating cytokine production and cellular crosstalk. The reversibility and specificity of many PTMs position them as attractive therapeutic targets to modulate antitumor immunity.

This review summarizes current insights into PTMs in colorectal cancer immunity. We outline major PTMs and their roles in shaping tumor immunogenicity, antigen presentation, and immune cell behavior, and evaluate their impact on immunotherapy response and resistance. We also explore emerging strategies to target PTM enzymes and leverage multi-omics to discover new biomarkers and therapeutic targets, aiming to improve outcomes in CRC immunotherapy.

## Major types and mechanisms of post-translational modifications in CRC

PTMs dynamically modulate key signaling pathways, protein stability, and epigenetic landscapes, thereby orchestrating tumor progression and immune evasion. In CRC, several PTMs have emerged as pivotal regulators of both intrinsic tumor biology and extrinsic immune responses.

### Phosphorylation governs tumor progression and therapy resistance in CRC

**Fig. 1:**
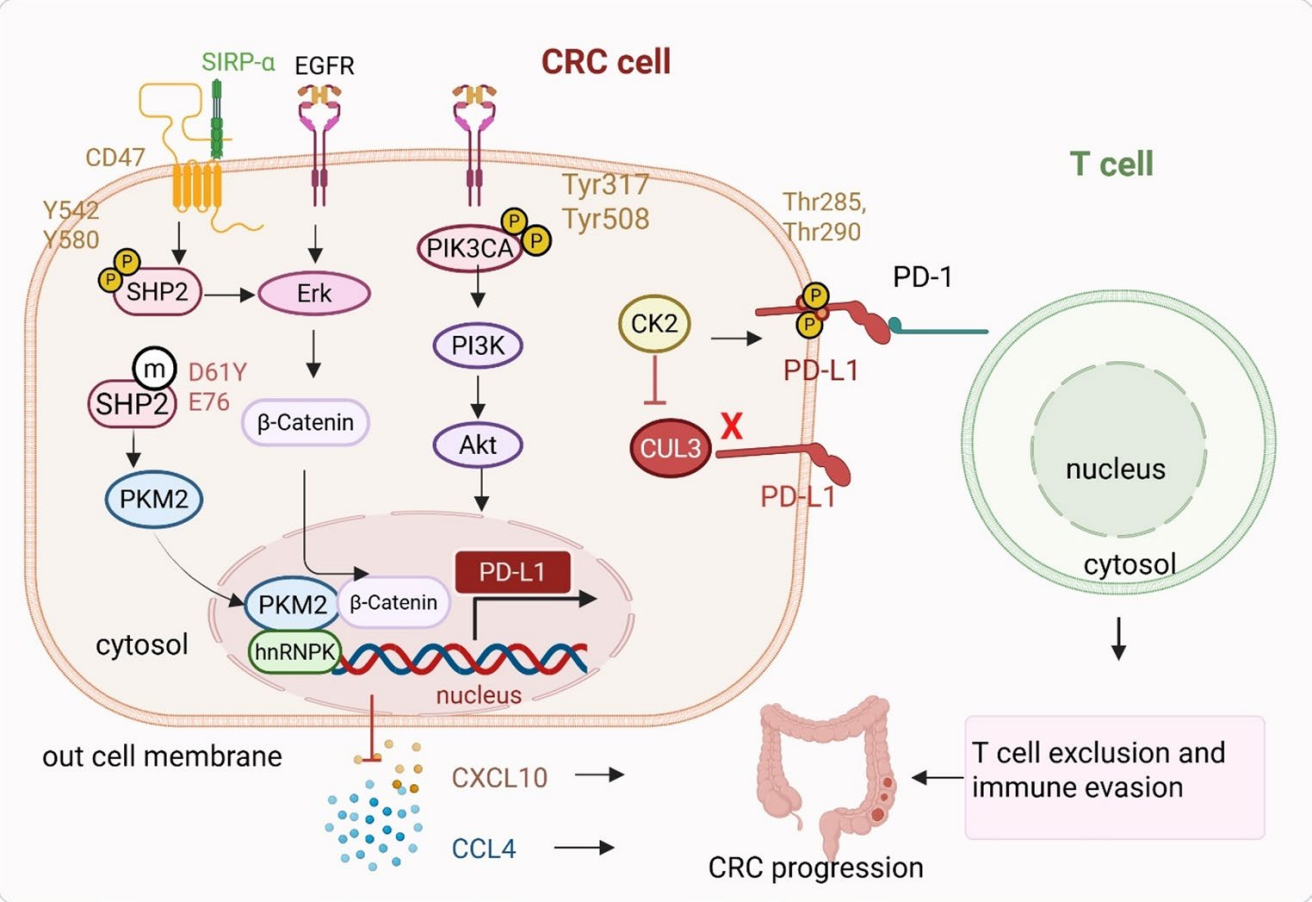
Phosphorylation-mediated regulation of immune evasion and metabolic adaptation in CRC. Phosphorylation stabilizes immune checkpoint proteins and modulates signaling pathways to promote tumor immune escape and metabolic reprogramming. CK2 phosphorylates PD-L1 at Thr285 and Thr290, preventing its ubiquitination and degradation, thereby maintaining high PD-L1 levels on tumor and dendritic cells and suppressing T cell activity via PD-1 interaction. PIK3CA phosphorylation at Tyr317 and Tyr508 activates PI3K/AKT signaling, enhancing PD-L1 transcription and dampening cytotoxic T cell responses. The phosphatase SHP2 sustains ERK and β-catenin signaling, suppressing chemokines (CCL4, CXCL10) and promoting T cell exclusion. Oncogenic SHP2 mutations facilitate nuclear translocation of PKM2, which stabilizes hnRNPK to drive proliferation, migration, and chemotherapy resistance. These phosphorylation events collectively integrate oncogenic, immune, and metabolic pathways, supporting CRC progression and immune evasion

Phosphorylation, a reversible post-translational modification mediated by kinases and phosphatases, plays a central role in coordinating oncogenic pathways, immune evasion, metabolic plasticity, and therapy resistance in CRC ([14]– [15]) (Fig. 1).This dynamic regulatory mechanism integrates signals across tumor-intrinsic and microenvironmental contexts, enabling tumor cells to adapt to immune surveillance and therapeutic stress.

#### Phosphorylation coordinates immune evasion and metabolic adaptation

In the immune system, phosphorylation promotes tumor immune evasion by stabilizing immune checkpoint molecules and suppressing antitumor signaling. For instance, CK2-mediated phosphorylation of PD-L1 at Thr285 and Thr290 prevents its binding to the CUL3 ubiquitin ligase adaptor, thereby inhibiting proteasomal degradation and maintaining high PD-L1 expression in tumor and dendritic cells [16]. Similarly, phosphorylation of PIK3CA (p110α) at Tyr317 and Tyr508 enhances PI3K/AKT signaling, upregulating PD-L1 transcription and weakening T cell-mediated cytotoxicity [17]. Phosphatases such as SHP2 (PTPN11) function as key immune regulators by sustaining ERK and β-catenin activation, leading to T-cell exclusion through suppression of chemokines like CCL4 and CXCL10 [18]. SHP2 also modulates innate immunity in microsatellite-stable (MSS) tumors, where its inhibition activates STING-TBK1-IRF3 signaling in myeloid cells and restores type I interferon responses [19]. SHP2 activity itself is regulated by neddylation and CD47-SIRPα interactions, which govern its autoinhibitory state and phagocytosis-suppressing function at the macrophage surface [20]. Notably, oncogenic SHP2 mutations (e.g., SHP2D61Y and SHP2E76K) drive CRC (HCT116 and HT-29 cell modal) progression by promoting glycolysis through nuclear translocation of PKM2, which stabilizes hnRNPK and enhances proliferation, migration, and cisplatin resistance [21].

#### Phosphorylation enhances tumor proliferation and drives therapeutic resistance

Beyond immune regulation, phosphorylation also orchestrates CRC cell proliferation and translation through substrate-specific phosphorylation. CDK15 phosphorylates PAK4 at Ser291, stimulating β-catenin/c-Myc and MEK/ERK signaling [22]; CDK13 promotes MYC-dependent protein synthesis by targeting translation factors 4E-BP1 (Thr46) and eIF4B (Ser422), with dual inhibition of CDK13 and mTORC1 exhibiting synergistic antitumor effects [23]. Similarly, under ferroptotic stress, CDK1 phosphorylates NANS at Ser275, triggering UBE2N-mediated ubiquitination and degradation, which in turn activates TAK1-NF-κB signaling and FTH1 expression, facilitating ferroptosis resistance and metastasis [24]. Additionally, phosphorylated STAT3 in cancer-associated fibroblasts (CAFs) drives angiogenesis and tumor growth, and phospho-STAT6 supports M2 macrophage polarization through JAK1 signaling, emphasizing the immunoregulatory impact of phosphorylation in the tumor microenvironment [25].

Phosphorylation also modulates therapeutic resistance. For instance, phosphorylation of NFS1 at Ser293 prevents PANoptosis and contributes to oxaliplatin resistance [26]. Similarly, chemotherapy-induced activation of MAPK signaling leads to ERK-mediated phosphorylation of RFNG at Ser255, promoting its nuclear translocation and inhibition of CHK2-mediated p53 phosphorylation, thereby suppressing apoptosis and ferroptosis [27]. RIOK1 enhances radioresistance via phosphorylation of G3BP2 at Thr226, which stabilizes MDM2 and promotes p53 degradation [28]. In parallel, IL-6-induced BECN1 phosphorylation (Tyr333) by JAK2 promotes autophagy by promoting PI3KC3 complex formation and chemoresistance [29], while phosphorylation of PPDPF (Tyr16/17) links inflammation to Wnt activation via disassembly of the β-catenin destruction complex [30]. Recent phosphoproteomic profiling highlights additional phosphorylation-based mechanisms in CRC progression and drug response. For example, resistance to EGFR blockade is associated with elevated MAPK and AKT phosphorylation and epithelial-mesenchymal transition (EMT) features, while sensitivity correlates with tyrosine phosphorylation of junction proteins (e.g., CXADR, CLDN3). Kinases like Src and ephrin family members emerged as alternative therapeutic targets in cetuximab-resistant models [31]. Moreover, destabilization of TRAF6 by GSK3β-driven phosphorylation (Thr266) impairs selective autophagic degradation of β-catenin and promotes EMT [32]. Phosphorylation status also affects transcriptional regulation, such as PLK1-mediated Ser326 phosphorylation of OTUD3, which stabilizes YY1 and supports tumor growth [33], or the downregulation of PCYT2, which reduces YAP1 phosphorylation, enhancing metastasis through SNAIL2 and ZEB1 activation [34].

Together, these findings underscore the multifaceted role of phosphorylation in colorectal cancer by integrating oncogenic, immune, and metabolic signals. Deciphering these phosphorylation-dependent networks not only expands our understanding of CRC pathogenesis but also reveals novel kinase targets and combinatorial strategies for enhancing immunotherapy and overcoming drug resistance.

### Ubiquitination and deubiquitination govern protein fate and shape tumor immunity in CRC

**Fig. 2:**
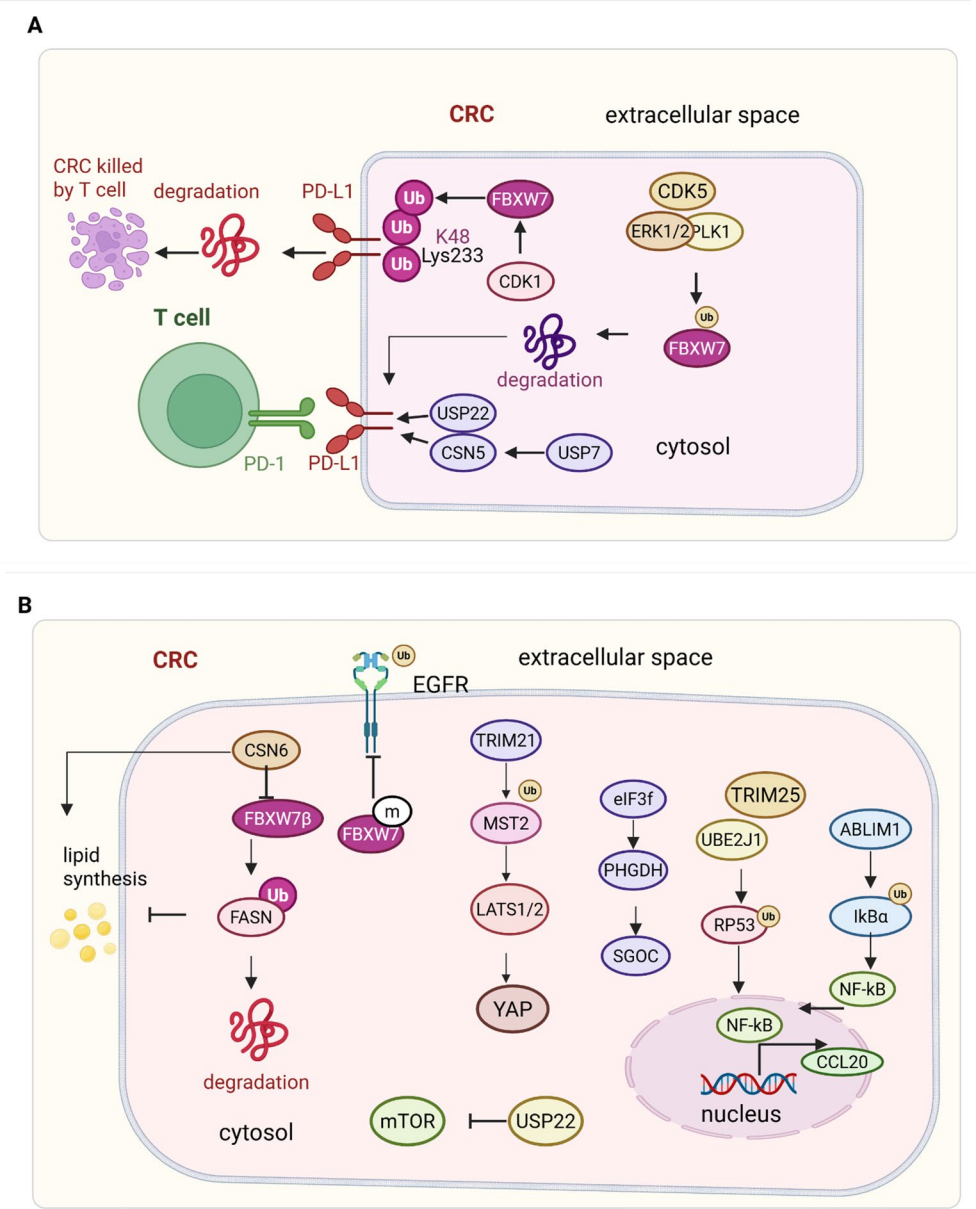
Ubiquitin signaling orchestrates immune regulation and metabolic reprogramming in colorectal cancer (CRC). (**A**) Immune checkpoint modulation: The E3 ligase FBXW7 promotes K48-linked ubiquitination of PD-L1 at Lys233 in a CDK1-dependent manner, facilitating its proteasomal degradation and enhancing antitumor immunity. FBXW7 is negatively regulated by phosphorylation (via PLK1, ERK1/2, and CDK5), which induces its autoubiquitination and degradation, resulting in accumulation of oncogenic substrates. Conversely, USP22 directly stabilizes PD-L1 by removing multiple ubiquitin linkages (K6, K11, K27, K29, K33, K63), while USP7 indirectly maintains PD-L1 stability by deubiquitinating CSN5, promoting immune evasion. (**B**) Metabolic and signaling control: FBXW7β ubiquitinates and degrades FASN and PHGDH, suppressing lipogenesis and the serine–glycine–one-carbon (SGOC) pathway; however, CSN6 destabilizes FBXW7β, restoring metabolic flux. The deubiquitinase eIF3f stabilizes MYC and PHGDH to support metabolic reprogramming. Loss of USP22 enhances mTOR signaling and tumor growth. UBE2J1, in complex with TRIM25, promotes RPS3 ubiquitination and activates NF-κB signaling. ABLIM1 mediates IκBα degradation, triggering NF-κB–dependent CCL20 expression and tumor invasion. In contrast, TRIM21 catalyzes K63-linked ubiquitination of MST2, activating the Hippo pathway and inactivating YAP, thereby suppressing metastasis

Ubiquitination is a reversible post-translational modification involving the covalent attachment of ubiquitin molecules to lysine residues on substrate proteins, orchestrated by a cascade of E1 activating, E2 conjugating, and E3 ligating enzymes, while deubiquitinating enzymes (DUBs) remove ubiquitin chains to reverse this modification [35–37]. In CRC, the dynamic balance between ubiquitination and deubiquitination critically governs the stability of oncogenes, tumor suppressors, and immune checkpoint proteins, thereby shaping tumor progression and immune evasion (Fig. 2).

#### Ubiquitination regulates protein stability and immune checkpoint expression in CRC

Ubiquitination and deubiquitination play critical roles in shaping the immune landscape of CRC by dynamically controlling the stability of immune checkpoint proteins, tumor suppressors, and oncogenes. FBXW7, an E3 ubiquitin ligase frequently mutated in ~ 16% of CRC cases, targets PD-1 for K48-linked ubiquitination at Lys233 in a CDK1-dependent manner, promoting its proteasomal degradation and thereby enhancing antitumor immunity; loss of FBXW7 stabilizes PD-1 and confers resistance to PD-1 blockade [38]. FBXW7 is itself tightly regulated through phosphorylation by PLK1 (Thr205), ERK1/2, and CDK5 (Ser349/372), which promotes its autoubiquitination and degradation, resulting in the accumulation of oncogenic substrates such as c-Myc and Notch1 and leading to impaired immune surveillance [39]. On the deubiquitination side, USP22 stabilizes PD-L1 by removing multiple ubiquitin linkages (K6, K11, K27, K29, K33, K63); its expression is upregulated by EZH2 inhibition, enhancing PD-L1 accumulation and immune escape [40]. Small-molecule targeting USP22 by demethylzeylasteral reactivates PD-L1 degradation, boosts CD8⁺ T cell function, and synergizes with CTLA-4 blockade in vivo [41]. Additionally, USP7 contributes to PD-L1 stability indirectly through deubiquitination of CSN5, which itself deubiquitinates PD-L1, suggesting that USP7 inhibition could restore T cell-mediated antitumor responses [42]. Beyond immune checkpoints, USP22 also acts within the SAGA complex to regulate H2B ubiquitination, transcriptional control, and metastasis in APC-mutant mouse models, demonstrating a context-dependent dual role in CRC [43]. Collectively, these findings illustrate that ubiquitin signaling controls immune checkpoint availability and transcriptional programs, offering therapeutic opportunities for immune modulation in CRC.

#### Ubiquitination controls metabolism and signaling to promote tumor growth and therapy resistance

Ubiquitin-dependent mechanisms tightly regulate key metabolic enzymes and signaling pathways that fuel colorectal tumor progression and confer resistance to therapy. FBXW7β targets fatty acid synthase (FASN) for degradation, but CRC-specific mutations disrupt this process and sustain lipogenesis. The oncogenic protein CSN6 binds FBXW7β, enhances its autoubiquitination and degradation, and stabilizes FASN, thereby activating the EGF–CSN6–FASN axis to promote tumor growth [44]. Similarly, FBXW7 mediates EGFR degradation via phosphodegron motifs, but mutations in FBXW7 or within these EGFR motifs prevent its ubiquitination, reduce EGF dependency, and induce resistance to EGFR–MAPK inhibitors [45]. In the metabolic arm, FBXW7β also targets phosphoglycerate dehydrogenase (PHGDH) for degradation; however, EGF signaling suppresses this function via GSK3β inactivation. Concurrently, eIF3f acts as a deubiquitinase that stabilizes PHGDH and MYC, activating the serine-glycine-one-carbon (SGOC) pathway to support CRC proliferation [46]. USP22 also regulates mTOR signaling, and its loss in CRC enhances mTOR activity and tumor burden, further underscoring the diverse context-dependent outcomes of DUB modulation [43]. UBE2J1, an E2 conjugating enzyme silenced by promoter hypermethylation in CRC, forms a complex with TRIM25 to ubiquitinate RPS3. Loss of UBE2J1 stabilizes RPS3, activates NF-κB signaling, and promotes tumor proliferation and metastasis [47]. Likewise, ABLIM1 functions as a novel E3 ligase for IκBα, targeting it for proteasomal degradation and activating downstream NF-κB/CCL20 signaling, which drives CRC cell invasion and progression [48]. In contrast, TRIM21 acts as a metastasis suppressor by promoting K63-linked ubiquitination of MST2 at Lys473, enhancing MST2 dimerization and kinase activity, leading to YAP inhibition and suppression of epithelial–mesenchymal transition. Vilazodone, an antidepressant, directly activates TRIM21 and exerts anti-metastatic effects both in vitro and in vivo [7]. These findings emphasize that ubiquitin-dependent regulation of signaling and metabolism directly dictates CRC growth dynamics, metastatic behavior, and drug resistance.

#### Ubiquitination modulates ferroptosis, immune evasion, and chemoresistance in CRC

Ubiquitin signaling also governs ferroptosis, immunosuppressive signaling, and chemoresistance in CRC through diverse pathways. The deubiquitinase USP11 stabilizes LSH, a chromatin remodeler that promotes CYP24A1 transcription. CYP24A1, in turn, limits intracellular calcium influx and lipid peroxidation, conferring ferroptosis resistance and facilitating tumor survival under oxidative stress [49]. USP14 stabilizes IDO1, an enzyme that promotes tryptophan metabolism and suppresses T-cell function; its inhibition restores cytotoxic T cell activity and enhances anti–PD-1 responses without activating the aryl hydrocarbon receptor (AhR) pathway [50]. USP21 also plays a central role in immune evasion by deubiquitinating and stabilizing both PD-L1 and Foxp3, enhancing regulatory T cell (Treg) function. Gallic acid downregulates USP21 transcription by inhibiting STAT3 phosphorylation, thereby promoting the degradation of PD-L1 and Foxp3 and amplifying the efficacy of PD-1 blockade [51]. Beyond proteins, non-coding RNAs such as circSEC24B integrate into the ubiquitin regulatory network by acting as scaffolds; circSEC24B facilitates OTUB1-mediated deubiquitination of SRPX2, enhancing its stability and promoting autophagy via acetylation-dependent mechanisms. This regulation ultimately contributes to chemoresistance and tumor progression in CRC [52]. Together, these examples highlight how ubiquitin regulation coordinates immune tolerance, stress resistance, and therapy responsiveness through both protein- and RNA-based networks.

In summary, ubiquitination and deubiquitination represent central molecular switches that regulate key biological processes in colorectal cancer, including immune checkpoint stability, oncogenic signaling, metabolic reprogramming, ferroptosis, and chemoresistance. Disruption of this finely tuned system contributes to tumor growth, immune escape, and therapy resistance. Targeting specific E3 ligases or deubiquitinases, or modulating ubiquitin interactions through RNA scaffolds or small molecules, offers promising therapeutic strategies for precision immunotherapy and metabolic intervention in CRC.

### Acetylation and deacetylation regulate immune responses and remodel tumor microenvironment in CRC

**Fig. 3:**
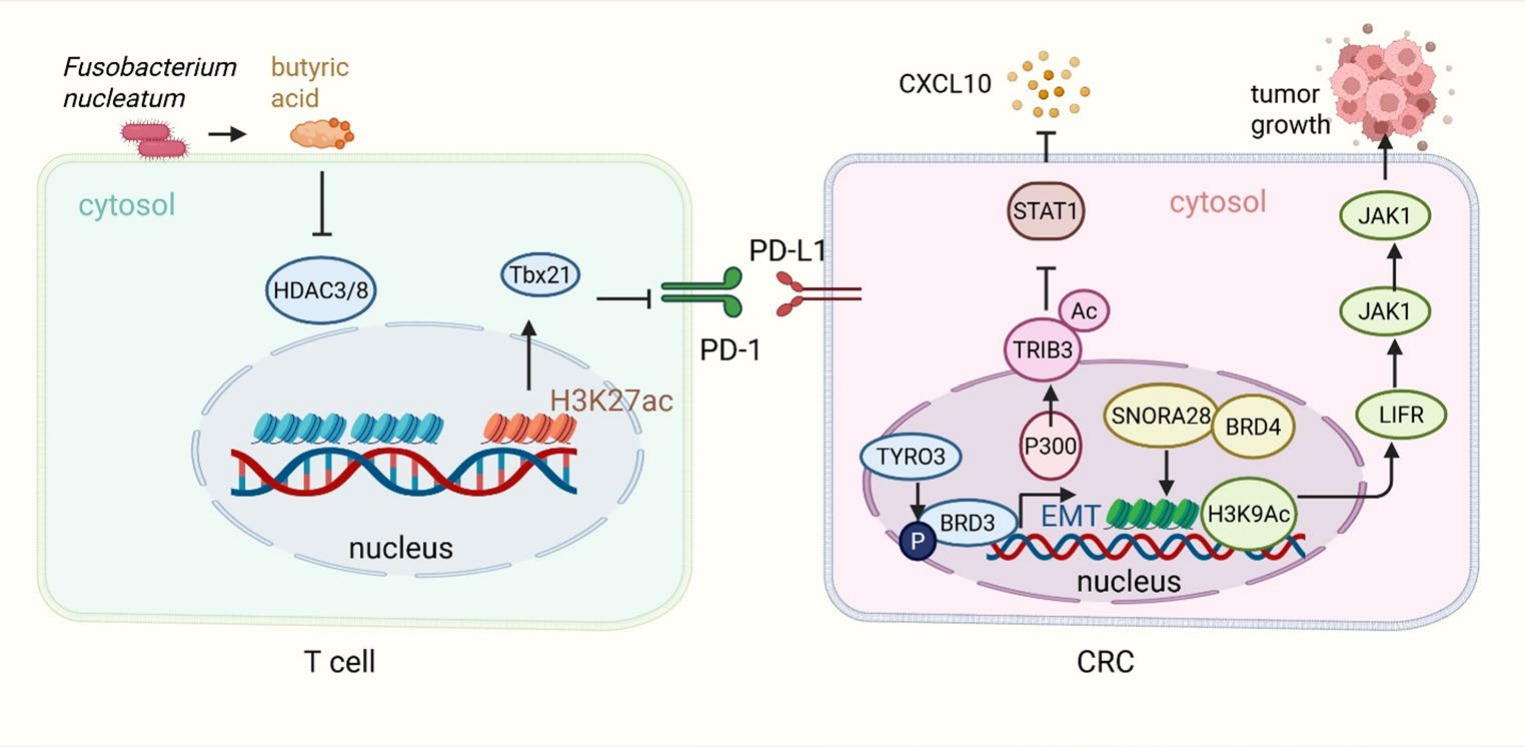
Acetylation and deacetylation regulate immune responses and remodel the tumor microenvironment in CRC. (**A**) Immune regulation and immunotherapy response: In CD8⁺ T cells, butyric acid derived from *Fusobacterium nucleatum* inhibits HDAC3/HDAC8, increases H3K27 acetylation at the *Tbx21* promoter, enhances TBX21 expression, reduces PD-1 expression, and limits T cell exhaustion, thereby improving anti–PD-1 therapy efficacy. In CRC cells, P300-mediated acetylation stabilizes TRIB3, which suppresses STAT1 activation and CXCL10 expression, decreasing CD8⁺ T cell infiltration. SNORA28 recruits BRD4 to increase H3K9 acetylation at the *LIFR* promoter, activating the JAK1/STAT3 pathway and promoting proliferation. TYRO3 phosphorylates BRD3, which epigenetically drives EMT and anti-apoptotic genes

Acetylation, typically occurring on lysine residues, is a reversible post-translational modification that profoundly influences chromatin structure and protein function. Histone acetyltransferases (HATs) catalyze the addition of acetyl groups, loosening chromatin and promoting gene transcription, while histone deacetylases (HDACs) remove acetyl groups, generally leading to transcriptional repression. Beyond histones, acetylation of non-histone proteins modulates protein stability, localization, and interaction networks [53]. In CRC, the dynamic balance between acetylation and deacetylation regulates key immune-related genes and signaling pathways, shaping the tumor microenvironment and response to immunotherapy (Fig. 3).

#### Acetylation modulates immune regulation and therapy response in CRC

Acetylation and deacetylation dynamically regulate immune responses in CRC by epigenetically controlling gene expression and signaling pathways that shape tumor-immune interactions. In CRC, particularly microsatellite stable (MSS) subtypes, histone acetylation influences T cell function and immunotherapy outcomes. For example, *Fusobacterium nucleatum* produces butyric acid, which inhibits HDAC3 and HDAC8 in CD8^+^ T cells, increasing H3K27 acetylation at the *Tbx21* promoter. This epigenetic change elevates TBX21 expression, suppresses PD-1 transcription, reduces T cell exhaustion, and enhances anti-PD-1 immunotherapy efficacy [42]. Similarly, acetylation stabilizes the oncoprotein TRIB3 via P300-mediated modification, preventing its degradation and resulting in suppression of STAT1 activation and CXCL10 expression, thereby decreasing CD8^+^ T cell infiltration. Pharmacological targeting of this acetylation process restores T cell recruitment and sensitizes tumors to immune checkpoint blockade [54].

Non-histone protein acetylation also contributes to immune regulation by modulating tumor cell signaling. SNORA28 facilitates radioresistance by recruiting BRD4 to increase H3K9 acetylation at the *LIFR* promoter, which activates the JAK1/STAT3 pathway and promotes tumor cell proliferation [55]. Furthermore, the acetyl-lysine reader BRD3, phosphorylated by nuclear TYRO3, epigenetically regulates anti-apoptotic and epithelial-mesenchymal transition genes, driving CRC metastasis. Inhibiting this MMP-2/TYRO3/BRD3 axis disrupts acetylation-dependent signaling and reduces tumor progression [56]. Collectively, these findings underscore the pivotal role of acetylation in orchestrating immune evasion and therapy resistance in CRC.

#### Acetylation drives metabolic reprogramming and microenvironment remodeling in CRC

Acetylation critically modulates metabolic enzymes and signaling pathways that reprogram tumor metabolism and remodel the CRC microenvironment, promoting tumor progression and metastasis. For instance, acetylation of IDH1, mediated by disrupted interaction with SIRT1, induces mitochondrial oxidative stress and dysfunction, representing a potential vulnerability for therapeutic intervention [57]. Similarly, acetylation of vinculin by acetic acid derived from the microbiota *Alcaligenes faecalis* impairs its binding to β-catenin, disrupting the intestinal barrier and facilitating inflammation-driven CRC development [58]. Metabolically, acetylation regulates key enzymes and signaling hubs. ENO2-derived phosphoenolpyruvate inhibits HDAC1, increasing β-catenin acetylation and activating its oncogenic pathway, contributing to resistance against antiangiogenic therapy [59]. PINK1 suppresses CRC progression by promoting mitophagy and reducing acetyl-CoA production; loss of PINK1 leads to elevated acetyl-CoA and enhanced tumor growth through epigenetic and metabolic shifts [60]. The acetyltransferase NAT10 enhances mRNA stability of *KIF23* via ac4C RNA modification, thereby activating Wnt/β-catenin signaling to promote tumorigenesis [61].

Histone acetyltransferases like KAT7 facilitate CRC progression by acetylating histone H3 at lysine 14, upregulating *MRAS* transcription and activating the MAPK/ERK pathway [62], while SIRT1-mediated β-catenin deacetylation regulates metabolic switching from glycolysis to fatty acid oxidation under glucose deprivation, supporting tumor survival [63]. Additionally, acetylation at specific residues modulates protein stability, as shown by GCN5-mediated acetylation of DACH1 at lysine 680, which enhances its interaction with USP7, protecting DACH1 from ubiquitin-mediated degradation and promoting aggressive CRC phenotypes [64].

### Methylation in chromatin remodeling and immune suppression in colorectal cancer

**Fig. 4:**
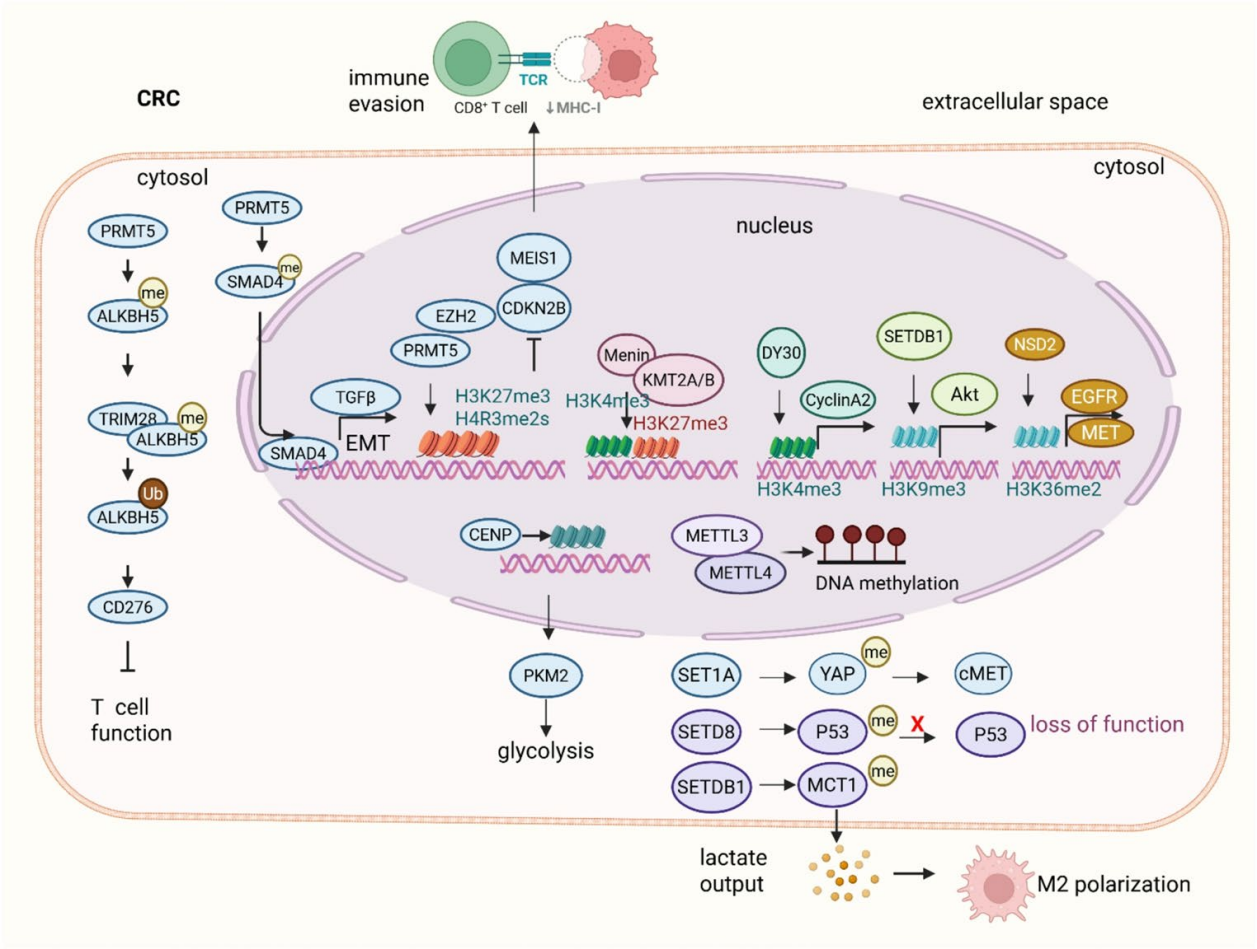
Methylation-mediated chromatin remodeling, metabolic reprogramming, and immune evasion in CRC. Methylation dynamically regulates histone and non-histone proteins, shaping oncogenic transcription, metabolic adaptation, cancer stemness, and immune evasion in CRC. SETDB1 promotes tumor glycolysis and M2 macrophage polarization by catalyzing H3K9 trimethylation and methylating MCT1 at lysine 473. Loss of ABHD5 induces DPY30 nuclear translocation and SET1A activation, which methylates YAP and histone H3 to upregulate c-Met transcription, reinforcing CRC stemness. NSD2 enhances H3K36me2 levels, activating Akt signaling via upregulation of EGFR and MET. At the transcriptional level, PRMT5 and EZH2 cooperatively deposit repressive histone marks (H4R3me2s, H3R8me2s, H3K27me3) at the CDKN2B promoter, leading to CpG island hypermethylation and gene silencing. SETD8-mediated mono-methylation of p53 at lysine 382 inactivates p53 in CRC stem cells, facilitating tumor progression. Epigenetic regulation intersects with metabolism: HDAC2 suppresses ALKBH5 via H3K27 deacetylation, resulting in excessive m6A RNA methylation, stabilization of JMJD8, and PKM2-driven glycolysis. The METTL3/METTL14 complex installs m6A modifications to recruit DNMT1, linking RNA and DNA methylation in oncogenic transcriptional control. In immune evasion, PRMT5 methylates ALKBH5 at R316, promoting its ubiquitination and degradation via TRIM28. Loss of ALKBH5 reduces m6A demethylation of CD276 mRNA, leading to elevated CD276 expression and suppression of cytotoxic T cell activity, thereby contributing to immune escape

Methylation, particularly lysine and arginine methylation of histones and methylation of non-histone proteins, constitutes a crucial post-translational modification regulating chromatin structure and gene expression. This modification is catalyzed by specific methyltransferases (“writers”) and reversed by demethylases (“erasers”). In CRC, aberrant methylation patterns profoundly influence immune gene regulation, tumor immune evasion, and therapeutic response (Fig. 4).

#### Methylation drives tumor proliferation, metabolic reprogramming, and stemness in CRC

Methylation of histones and non-histone proteins plays a fundamental role in regulating chromatin structure and gene expression, thereby influencing colorectal cancer cell proliferation, metabolic adaptation, and maintenance of cancer stemness. For instance, SETDB1-mediated tri-methylation of MCT1 at lysine 473 prevents its autophagic degradation, which enhances tumor glycolysis and promotes M2-like macrophage polarization by facilitating lactate shuttling, thus contributing to CRC progression [65]. This metabolic reprogramming is further exemplified by loss of ABHD5, which induces DPY30 nuclear translocation and promotes SET1A-mediated methylation of YAP and histone H3. This cascade enhances YAP-driven c-Met transcription, sustaining tumor stemness and growth [66, 67]. Similarly, histone H3R117 mono-ADP-ribosylation promotes IGFBP1 promoter methylation by increasing recruitment of H3K9me2, HP1, and DNMT1, thereby silencing tumor suppressor genes, disrupting lipid metabolism, and inhibiting apoptosis—key processes driving CRC development [68]. These epigenetic alterations converge on silencing critical tumor suppressors, as evidenced by PRMT5 and EZH2 cooperation, which deposits repressive methylation marks such as H4R3me2s, H3R8me2s, and H3K27me3 on the CDKN2B promoter, resulting in transcriptional repression and CpG hypermethylation that favor tumor cell proliferation [69].

The metabolic and proliferative advantages conferred by methylation are further reinforced by HDAC2-mediated H3K27 deacetylation, which suppresses ALKBH5 expression. This suppression leads to aberrant m6A RNA methylation that stabilizes JMJD8 and enhances PKM2 activity, promoting glycolysis and tumor progression, while restoration of ALKBH5 expression reverses these oncogenic effects [70]. In parallel, SETD8-mediated mono-methylation of p53 at lysine 382, induced by tumor-associated macrophages through IL-6/MCP-1 signaling, functionally inactivates p53 in CRC stem cells, facilitating immune evasion and tumor progression [71]. Such methylation-driven metabolic reprogramming also involves CENP-N–mediated lysine methylation of SEPT9, which increases glycolytic enzyme expression and aerobic glycolysis, promoting proliferation, migration, and liver metastasis [72]. NSD2 further advances these processes by catalyzing H3K36 dimethylation (H3K36me2), upregulating oncogenes like EGFR and MET, and activating Akt signaling, thus enhancing cancer cell survival and invasiveness [73].

Beyond histone methylation, RNA methylation acts synergistically to regulate gene expression in CRC. The METTL3-METTL14 complex installs m6A marks that recruit DNMT1, inducing DNA methylation at gene bodies to coordinately regulate transcription and mRNA stability, fine-tuning oncogenic and differentiation-related pathways [74]. Concurrently, UTX deficiency leads to stabilization of phenylalanine hydroxylase and increased tyrosine secretion, which promotes myeloid-derived suppressor cell (MDSC)-mediated immunosuppression and metastasis through STAT5 activation, exemplifying the crosstalk between metabolic and epigenetic regulation in CRC progression [75].Moreover, SETDB1-mediated H3K9 methylation activates Akt signaling preferentially in KRAS-mutant CRC, contributing to tumorigenicity and chemoresistance, positioning SETDB1 as a potential therapeutic target [76].

#### Methylation shapes immune evasion, chemoresistance, and epigenetic dysregulation in CRC

In addition to promoting tumor growth and metabolism, methylation plays a decisive role in CRC immune evasion and resistance to chemotherapy. The m6A methyltransferase ZCCHC4 is notably upregulated in CRC, where it suppresses the lncRNA GHRLOS, subsequently regulating KDM5D to enhance cell proliferation, migration, and invasion, underscoring RNA methylation’s pivotal role in tumor progression [77]. This theme of transcriptional activation is echoed by DPY30, which promotes H3K4 trimethylation at key proliferation-associated gene promoters—including PCNA, Ki67, and Cyclin A2—thereby driving tumor cell cycle progression [78].

Histone crotonylation also contributes to CRC aggressiveness: increased H3K27 crotonylation in metastatic CRC promotes invasion through LINC00922-mediated inhibition of SIRT3 binding, which activates ETS1 transcription [79]. Along similar lines, CREB-binding protein (CBP)/p300-mediated H3K27 acetylation upregulates Timeless expression, facilitating β-catenin nuclear translocation via myosin-9 interaction and promoting tumorigenesis [80]. These epigenetic activations are counterbalanced by oncogenic metabolites such as R-2-hydroxyglutarate (R-2HG), produced by mutant IDH1/2, which inhibits the αKG-dependent demethylase KDM4A. This inhibition increases H3K9me3 at telomeres, leading to telomeric dysfunction and genomic instability that drive tumorigenesis [81].

Moreover, metabolic products like β-hydroxybutyrate (BHB) modulate histone methylation to influence drug resistance; BHB suppresses H3K79 methylation to resensitize CRC cells to oxaliplatin, thereby inhibiting proliferation, migration, and epithelial-mesenchymal transition (EMT) [82]. The balance of methylation marks is critical, as downregulation of histone demethylase UTX—mediated by the CUL4B-DDB1-COP1 ubiquitin ligase complex—increases H3K27 methylation and promotes tumorigenesis, which can be partially reversed by inhibiting EZH2 methyltransferase [83]. At the genomic level, germline mutations in homologous recombination repair and DNA damage checkpoint genes (BRCA1/2, ATM, CHEK2) interact synergistically with somatic epigenetic alterations such as KDM6A mutations and ERBB2 amplification, collectively disrupting chromatin regulation and genome stability to drive CRC development [84]. Targeting the histone demethylases KDM6A/B reshapes the epigenetic landscape to suppress colorectal tumor-initiating cell (TIC) properties, diminishing stemness gene expression and enhancing chemosensitivity [85]. Epigenetic repression of MEIS1 by the EZH2-DNMT3a complex, facilitated by lncRNA ELFN1-AS1, also mediates oxaliplatin resistance by enhancing DNA damage repair, and disrupting this methylation-dependent axis restores drug sensitivity [86]. The Menin-KMT2A/B complex maintains a fine balance between activating H3K4me3 and repressive H3K27me3 at bivalent promoters; loss or inhibition of Menin disrupts this equilibrium, leading to aberrant gene activation and CRC progression [87]. Furthermore, TRIM25 promotes oxaliplatin resistance by stabilizing EZH2 through inhibition of its ubiquitination, enhancing EZH2-driven histone methylation and fostering cancer stemness [88].

Crucially, beyond these mechanisms of epigenetic dysregulation and chemoresistance, emerging evidence demonstrates that DNA methylation and RNA methylation cooperatively shape the tumor immune microenvironment in CRC, thereby facilitating immune evasion. In particular, recent reviews highlight that aberrant DNA methylation of tumor-suppressor genes, antigen-presentation machinery, and interferon-signaling components leads to reduced effector T-cell infiltration, impaired neoantigen presentation, and a “cold” immune-excluded phenotype in solid tumors including colorectal cancer [89]. For instance, silencing of the STING pathway via promoter hypermethylation reduces type I interferon responses and thus weakens antitumor immunity [89]. Moreover, a recent colon-cancer cohort study found that a high DNA-methylation gene signature correlates with lower CD8⁺ T cell and NK cell infiltration and worse prognosis [90]. These findings underscore the dual role of methylation: not only driving intrinsic tumor cell survival and chemoresistance, but also reprogramming the immune microenvironment to favour immune escape.

#### Methylation modulates signaling pathways and immune escape in CRC progression

Beyond tumor growth and therapy resistance, methylation tightly regulates critical signaling pathways and immune evasion mechanisms essential for CRC progression. PRMT5 methylates SMAD4 at arginine 361, a modification that enhances SMAD complex assembly and nuclear translocation, thereby activating TGF-β signaling. This activation drives EMT and metastasis, underscoring the role of methylation in tumor invasion and dissemination [91]. In parallel, PRMT5 catalyzes symmetric dimethylation of ALKBH5 at R316, which facilitates its ubiquitination and degradation by TRIM28. The consequent reduction in ALKBH5-mediated m6A demethylation on CD276 mRNA increases CD276 expression, facilitating immune evasion by suppressing cytotoxic T-cell activity and promoting tumor progression [92]. These mechanisms highlight how methylation-mediated regulation of signaling and immune checkpoints promotes an immunosuppressive tumor microenvironment, identifying methyltransferases such as PRMT5 as promising therapeutic targets to improve CRC outcomes.

Beyond classical histone and non-histone protein methylation, emerging evidence highlights the critical role of RNA methylation and metabolic-epigenetic crosstalk in colorectal cancer. For instance, m6A RNA modification installed by the METTL3-METTL14 complex can recruit DNA methyltransferase DNMT1 to coordinate DNA methylation and transcriptional regulation, fine-tuning oncogenic pathways [74]. Additionally, metabolic alterations such as UTX deficiency-driven tyrosine secretion promote immunosuppressive microenvironments via epigenetic mechanisms, exemplifying the intricate interplay between cellular metabolism and PTMs in CRC progression [75]. These layers of regulation extend the complexity of PTM-mediated chromatin remodeling and immune modulation, underscoring novel avenues for therapeutic intervention beyond traditional protein methylation targets74.

In summary, methylation-mediated modifications orchestrate a complex network of chromatin remodeling, metabolic reprogramming, and immune evasion that collectively drive colorectal cancer progression and therapeutic resistance. Targeting key methyltransferases and demethylases within these pathways offers promising strategies to reverse epigenetic dysregulation, overcome drug resistance, and enhance anti-tumor immunity in CRC.

### Glycosylation orchestrates immune evasion, metabolic reprogramming, and tumor progression in CRC

Glycosylation, a critical post-translational modification involving the enzymatic addition of carbohydrate moieties to proteins or lipids, plays multifaceted roles in modulating receptor stability, ligand-receptor interactions, immune surveillance, and cellular metabolism. In CRC, aberrant glycosylation patterns—particularly N-linked glycosylation and O-GlcNAcylation—reprogram oncogenic signaling and promote immune evasion and chemoresistance.

#### Glycosylation modulates immune evasion and therapy resistance in CRC

Glycosylation critically influences immune escape and drug resistance in colorectal cancer. Aberrant N-glycosylation destabilizes IFNγRα by downregulating MGAT3, leading to proteasomal degradation and impaired IFN-γ signaling, which can be reversed by restoring MGAT3 or treatment with all-trans retinoic acid [93]. PD-L1 undergoes essential N-glycosylation modifications that protect it from GSK3β-mediated degradation, promoting immune checkpoint stability and suppression of cytotoxic T lymphocytes. Targeting PD-L1 glycosylation restores antitumor immunity and enhances immunotherapy efficacy [94]. Moreover, site-specific N-glycosylation of plasma carcinoembryonic antigen (CEA) alters with CRC progression, affecting its molecular behavior and clinical utility as a biomarker [95]. The expression of claudin-3, regulated through N-glycosylation-dependent receptor tyrosine kinase signaling involving EGFR and IGF1R, correlates with CRC malignancy and poor prognosis, especially in CMS2 and CMS3 molecular subtypes [96]. Additionally, O-GlcNAcylation mediated by OGT modifies key transcription factors and metabolic enzymes such as β-catenin and c-Myc, promoting Wnt signaling, metabolic reprogramming, and chemoresistance, highlighting its role in tumor progression and therapy failure.

#### Glycosylation drives metabolic reprogramming and tumor progression in CRC

Aberrant glycosylation in CRC drives tumor progression by modulating glycan biosynthesis and PI3K-Akt signaling pathways, influencing immune cell infiltration and patient prognosis. Glycosylation-related gene expression profiles also predict therapeutic responses, offering new targets for personalized CRC treatment [97]. Specific N-glycosylation modifications, such as at Asn263 of cathepsin D (CTSD) by DDOST and STT3B, enhance protease activity leading to metabolic dysregulation and liver metastasis [98]. Glycosphingolipid synthesis is upregulated by ECHS1-mediated activation of UDP-glucose ceramide glycosyltransferase (UGCG), which activates PI3K/Akt/mTOR signaling and mitochondrial dysfunction, thereby fostering tumor progression and drug resistance; pharmacological inhibition of this axis reverses these effects [94]. Glycosylation also affects key metabolic proteins such as APMAP, with site-specific N-glycosylation contributing to metabolic alterations supporting tumor growth [99]. Overexpression of MGAT5-catalyzed β1,6-GlcNAc-branched N-glycans stabilizes multiple receptors including TGF-βR, EGFR, and PD-L1 by inhibiting their degradation, sustaining EMT, angiogenesis, immune suppression, and correlating with advanced CRC stage and poor prognosis. Collectively, glycosylation acts as a pivotal driver integrating metabolic rewiring and malignant progression in colorectal cancer.

In summary, glycosylation exerts multifaceted roles in colorectal cancer by modulating immune escape mechanisms, metabolic pathways, and receptor stability, thereby promoting tumor progression and resistance to therapy. Targeting aberrant glycosylation processes represents a promising avenue to enhance immunotherapy efficacy and inhibit metastatic progression in CRC.

## Diverse PTMs orchestrate antigen presentation and immune evasion in CRC

PTMs critically regulate antigen presentation in CRC by modulating the expression, stability, and activity of antigen processing machinery (APM) components, including proteasomes, TAP transporters, and MHC class I/II molecules. These modifications influence how tumor-derived antigens are processed and presented, thereby shaping immune recognition and responsiveness to immunotherapy.

In CRC, ubiquitination plays a pivotal role in MHC class I heavy chain turnover. The E3 ligase MARCH9 ubiquitinates MHC-I molecules, targeting them for lysosomal degradation and reducing surface antigen presentation [100], Conversely, deubiquitinases such as USP8 remove ubiquitin chains, stabilizing MHC-I and enhancing antigen display [101]. This dynamic balance modulates tumor antigen visibility to cytotoxic T cells. Similarly, in CRC and other malignancies, the E3 ubiquitin ligase FBXO11 mediates ubiquitination and proteasomal degradation of CIITA—the master transcription factor driving MHC class II expression—leading to reduced MHC-II levels and impaired CD4⁺ T cell activation [102]. Additionally, the CtBP transcriptional complex represses MHC class II pathway genes at the transcriptional level, further suppressing antigen presentation [102]. These ubiquitin-dependent regulatory mechanisms collectively dampen tumor immune recognition and promote immune evasion, highlighting potential targets for therapeutic intervention to restore MHC expression and enhance antitumor immunity. Moreover, phosphorylation also mediates antigen presentation and colorectal cancer progression by modulating cytokine signaling pathways. Specifically, IL-11 activates STAT3 phosphorylation, which inhibits IFNγ-induced STAT1 phosphorylation, thereby downregulating MHC-I and CXCL9 expression in tumor cells and impairing CD8⁺ T cell infiltration—promoting immune evasion and tumor growth in CRC [103].

Epigenetic regulation profoundly influences antigen presentation in metastatic colorectal cancer (CRC). Specifically, liver metastatic CRC cells show reduced chromatin accessibility at MHC and interferon (IFN) response gene loci, limiting their transcriptional activation and impairing antigen presentation. This epigenetic silencing—linked to HNF4A-driven chromatin remodeling—contributes to immune evasion and poor response to immune checkpoint blockade in CRC liver metastases [104]. Histone acetylation, mediated by p300/CBP, promotes MHC-I antigen processing and presentation (AgPP) in cancer cells, enhancing recognition by cytotoxic T lymphocytes [105]. Chemotherapeutic agents like oxaliplatin and mitoxantrone induce this effect via NF-κB-driven transcriptional activation of AgPP genes, independently of IFN-γ signaling, thereby sensitizing MHC-I–low tumors to immune checkpoint blockade [105]. In addition to MHC-I regulation, epigenetic PTM also critically control tumor-specific MHC class II (tsMHC-II) expression in colorectal cancer by modulating IFN-γ signaling pathways. Notably, EZH2-mediated histone methylation at the CIITA promoter represses tsMHC-II induction, thereby impairing antigen presentation and tumor immune recognition [106]. Pharmacological targeting of these epigenetic marks can restore MHC-II expression and boost anti-tumor immunity. Furthermore, the histone methyltransferase WHSC1 plays a crucial role in colorectal cancer by promoting IFN-γ-induced MHC class I antigen presentation through direct interaction with NLRC5, enhancing antitumor immunity without affecting PD-L1 expression [107]. Loss of WHSC1 impairs MHC-I expression, weakens immune responses, and facilitates tumor progression, highlighting WHSC1 as a key mediator linking epigenetic modification to antigen presentation and immune checkpoint efficacy in CRC. Histone methylation by PRMT1 epigenetically suppresses IFN-γ-induced MHC-I expression in colorectal cancer by inhibiting STAT1 activation, leading to impaired CD8⁺ T cell-mediated immune responses [108]. Pharmacological inhibition or genetic knockout of PRMT1 restores STAT1 signaling and MHC-I levels, thereby enhancing anti-tumor immunity and improving responsiveness to immune checkpoint blockade.

In addition to ubiquitination, phosphorylation, and histone modifications, glycosylation also modulates antigen presentation. N-glycosylation regulates antigen presentation by modifying MHC-II-bound immunopeptides through extensive glycan trimming during lysosomal processing [109]. Specifically, both under-processed (oligomannosidic) and hyper-processed (chitobiose core, paucimannosidic) N-glycans are enriched on presented peptides, suggesting that remodelling of N-glycans is a key feature of MHC-II antigen processing and may influence immune surveillance by shaping peptide selection and loading [109]. O-GlcNAcylation regulates antigen presentation by modifying a small subset of HLA class I peptides, particularly those derived from nuclear proteins like transcription factors [110]. These O-GlcNAcylated peptides are preferentially presented by HLA-B*07:02, whose binding groove accommodates the consensus GlcNAcylation motif, suggesting that allele-specific presentation of PTM-modified peptides may shape immune recognition in both normal and cancers [110].

## Therapeutic opportunities: Targeting PTM enzymes to enhance immunotherapy

PTMs critically regulate tumor immune evasion mechanisms in CRC by modulating immune checkpoints, antigen presentation, cytokine signaling, and immune cell function. Targeting enzymes responsible for these PTMs represents a promising therapeutic avenue to potentiate immunotherapy efficacy, overcome resistance, and improve patient outcomes [111]. This section elaborates on the main classes of PTM enzymes as therapeutic targets, their inhibitors (Table 1), mechanisms of immune modulation, and current translational progress.

**Table 1:** Comprehensive overview of PTM-related enzymes and therapeutic agents targeting post-translational modifications to enhance immunotherapy in colorectal cancer

PTM class	Enzyme name	Small molecule/inhibitor/antibody	Mechanism of immune modulation (CRC focus)	Clinical status (CRC or other tumors)	Reference
Phosphorylation	GSK3β	CHIR99021	Phosphorylates PD-L1, leading to its degradation, enhancing immune recognition	Preclinical	[134]
Phosphorylation	CK2	CX-4945 (Silmitasertib)	Phosphorylates STAT3 and other substrates, modulating inflammatory TME signaling	Phase I/II trials in other tumors	[16]
Phosphorylation	PIM1 kinase	AZD1208	Modulates immune checkpoint pathways, influences T cell proliferation	Preclinical	[135]
Ubiquitination	USP22	Experimental USP22 inhibitors	Deubiquitinates and stabilizes PD-L1, promoting immune escape	Preclinical	[41]
Ubiquitination	USP7	P5091, FT671	Regulates Treg function, MDSCs, and immune checkpoint protein turnover	Early clinical trials	[64]
Ubiquitination	USP14	IU1	Influences proteasome-mediated degradation of immune regulators	Preclinical	[50]
Ubiquitination	FBXO22 (E3 ligase)	Experimental	Targets immune regulators for degradation, modulating tumor immunogenicity	Preclinical	[114]
Acetylation	HDAC1	Vorinostat, Panobinostat, Romidepsin	Enhances antigen presentation and reverses T cell exhaustion by chromatin remodeling	Approved (hematologic malignancies); trials in CRC	[59]
Acetylation	HDAC6	Ricolinostat (ACY-1215)	Modulates non-histone protein acetylation, affecting immune cell function	Phase I/II trials	[136]
Acetylation	SIRT1	EX-527 (Selisistat)	Regulates NF-κB acetylation, influencing inflammatory responses	Preclinical	[137]
Methylation	EZH2	Tazemetostat	Mediates histone H3K27 methylation, repressing immune genes and shaping TIME	Approved for lymphoma; CRC trials ongoing	[40]
Methylation	PRMT5	GSK3326595, EPZ015666	Regulates methylation of histones and non-histone proteins affecting T cell function	Phase I/II clinical trials	[69]
Glycosylation	STT3A/B	Tunicamycin (broad inhibitor), experimental agents	Reduces PD-L1 glycosylation, destabilizing it and enhancing T cell recognition	Preclinical	[138]
Glycosylation	FUT8	Experimental agents	Modulates core fucosylation of PD-L1, influencing immune checkpoint stability	Preclinical	[139]
Phosphatase	PTPN2	Experimental agents	Negatively regulates JAK-STAT pathway, influencing IFN-γ response and immune escape	Preclinical	[140]
Phosphatase	SHP2	SHP099	Modulates PD-1 signaling and cytokine pathways, impacting immune suppression	Early phase clinical trials	[20]
SUMOylation	SENP1	Experimental	Regulates immune signaling via SUMOylation modulation of transcription factors	Preclinical	[141]
Neddylation	NAE1	MLN4924 (Pevonedistat)	Inhibits neddylation, affecting NF-κB and immune gene expression	Early clinical trials	[142]

### Target ubiquitin-modifying enzymes to regulate immune checkpoints and antigen stability

Ubiquitin-modifying enzymes, including E3 ligases and deubiquitinases (DUBs), orchestrate the turnover and functional regulation of immune checkpoint proteins, antigen-processing components, and transcription factors critical to tumor immune evasion. In CRC, dysregulation of these enzymes contributes to immune suppression, therapeutic resistance, and tumor progression.

DUBs such as USP22 and USP7 have emerged as key immunomodulatory targets. USP22 stabilizes PD-L1 by removing K48-linked ubiquitin chains, sustaining its expression on the tumor cell surface and dampening CD8⁺ T cell-mediated cytotoxicity. Inhibition of USP7 with small molecules such as P5091 — which primarily targets USP7 rather than USP22 directly — promotes PD-L1 degradation and enhances responsiveness to PD-1 blockade, and may also indirectly affect USP22-mediated pathways to disrupt cancer stemness programs by downregulating transcription factors such as SOX2 and NANOG([42]– [43]). Similarly, USP7 supports regulatory T cell (Treg) function through stabilization of FOXP3 and modulates the MDM2–p53 axis in tumor cells. Its inhibitors (e.g., P22077, FT827) reprogram the tumor immune environment by weakening Treg-mediated suppression and restoring p53 activity, offering dual immune and tumor-intrinsic benefits [64–112]. USP14, a proteasome-associated DUB, regulates antigen processing and MHC class I expression. Its inhibitor VLX1570, currently in early-phase clinical evaluation (NCT02372240), enhances tumor antigenicity and may synergize with immune checkpoint blockade [113]. On the E3 ligase side, FBXO22 facilitates degradation of PD-L1 and promotes immune recognition, whereas CSN5 counteracts this process by deubiquitinating PD-L1, stabilizing it and supporting immune escape [114]. While these enzymes represent attractive targets, translating DUB and E3 ligase inhibitors into clinical practice faces challenges, including substrate specificity, on-target toxicity, and limited tumor selectivity. Emerging approaches such as proteolysis-targeting chimeras (PROTACs) and nanoparticle-mediated delivery systems offer promising solutions to improve therapeutic precision and safety in CRC.

### Inhibit kinases and phosphatases to modulate immune signaling pathways

Targeting phosphorylation regulators—kinases and phosphatases—offers a strategic avenue to reprogram immune signaling in CRC, enhance antigen presentation, and overcome resistance to immunotherapy.

Hyperactivation of the PI3K/AKT/mTOR axis is a common feature in CRC and contributes to both tumor progression and immune evasion. Recent preclinical models demonstrate that dual PI3K/mTOR inhibitors not only suppress CRC cell proliferation but also sensitize tumors to ferroptotic death pathways, presenting a multifaceted opportunity for immune modulation [115]. Selective PI3K inhibitors are now being evaluated in clinical settings, with biomarker-driven dosing strategies based on PIK3CA mutation status. These agents may restore interferon-driven antigen presentation and enhance CD8⁺ T cell recognition when combined with immune checkpoint blockade (ICB). On the phosphatase side, SHP2 represents a compelling target. SHP2 transmits inhibitory signals downstream of receptor tyrosine kinases and forms a key node in CRC immunoregulation. The oral SHP2 inhibitor RMC-4630 has demonstrated immunomodulatory effects in RAS-driven solid tumors, including CRC, in combination with MEK or KRAS^G12C^ inhibitors [116]. In preclinical models, SHP2 inhibition re-wires T cell signaling, potentiates interferon responses, and synergizes with PD-1 blockade to sustain durable tumor regression. Early-phase trials combining RMC-4630 with sotorasib and PD-1 inhibitors suggest feasibility and preliminary efficacy [117]. Efforts to target mTOR directly have also advanced. While traditional rapalogs primarily inhibit mTORC1, next-generation mTOR kinase inhibitors and dual PI3K/mTOR compounds—such as gedatolisib—block both mTORC1 and mTORC2 and are in early clinical testing for CRC([118]– [119]). These agents reduce survival signaling and may enhance MHC expression, though their immunomodulatory potential specifically in CRC remains under investigation.

Collectively, these kinase- and phosphatase-targeted approaches reprogram the tumor microenvironment by restoring interferon signaling, enhancing antigen presentation, and improving sensitivity to ICB. The integration of these agents into clinical protocols offers a rational strategy to convert immunologically “cold” CRC into responsive phenotypes.

### Block HDACs to restore immune gene expression and enhance antigen presentation

Histone deacetylases (HDACs) are key epigenetic regulators whose activity in CRC promotes transcriptional silencing of immune-related genes, including MHC-I/II, co-stimulatory molecules, and cytokines. Selective inhibition of HDACs restores chromatin accessibility and reactivates immune gene expression, improving tumor antigenicity and T cell recognition. Recent preclinical studies using the class I/II HDAC inhibitor zabadinostat (CXD101) demonstrated robust upregulation of MHC-I, MHC-II, and antigen processing machinery in CRC models, leading to increased CD8⁺ and CD4⁺ T cell infiltration. When combined with anti–PD-1 therapy, zabadinostat elicited pronounced tumor regression and durable immune memory in immunocompetent mouse models [120]. Moreover, a phase II randomized study (CAPability-01) in patients with chemo-refractory microsatellite-stable CRC showed that adding the HDACi Tucidinostat to PD-1 blockade and anti-VEGF therapy significantly increased intratumoral CD8⁺ T cell density and objective response rates compared to PD-1 inhibition alone [121]. Beyond boosting antigen presentation, HDAC inhibitors can alleviate immune checkpoints and reverse exhaustion. Pan-HDACi Vorinostat was found to suppress the HDAC1–JAK1–FGL1 axis, reduce T cell exhaustion markers in murine CRC models, and potentiate TIL cytotoxicity [122]. Another HDACi, Bocodepsin (OKI-179), induced transient histone hyperacetylation in peripheral CD4⁺/CD8⁺ T cells and enhanced T cell activation in vivo [123]. Remarkably, intermittent dosing during PD-1 blockade—rather than continuous administration—maximized antitumor efficacy, highlighting the importance of dosing schedules [123]. In addition, HDAC inhibitors also modulate immunosuppressive pathways. In CRC, the class I HDAC3 is implicated in regulating the expression of the inhibitory ligand B7x; HDAC3 inhibition leads to increased B7x expression, which may dampen antitumor immunity. Targeting B7x with neutralizing antibodies in combination with HDAC3 inhibitors reversed therapeutic resistance and enhanced CD8⁺ T cell infiltration in murine models [124]. Collectively, HDAC inhibition enhances CRC immunogenicity by restoring antigen presentation, reversing T cell exhaustion, and modulating the immunosuppressive microenvironment. These findings support the rationale for HDAC inhibitors as combinatorial partners with immunotherapy in CRC.

### Target protein methylation to reshape immune responsiveness

Protein methyltransferases (PMTs) have emerged as key modulators of antigen presentation in CRC, representing both inhibitory and stimulatory therapeutic targets. Among these, WHSC1, a histone H3K36 methyltransferase, promotes MHC class I expression and enhances tumor infiltration by CD8⁺ T cells, sensitizing CRC models to PD-1 blockade. Genetic ablation of WHSC1 impairs IFN-γ-induced antigen presentation, accelerates tumor growth in immune-competent hosts, and diminishes checkpoint efficacy, indicating that sustaining or activating WHSC1 may convert “cold” tumors into immunotherapy-responsive phenotypes [107]. In contrast, PRMT1, a type I arginine methyltransferase, restricts antigen presentation by suppressing STAT1-dependent MHC-I transcription. Knockout or inhibition of PRMT1 using the clinical-grade inhibitor GSK3368715 reverses this effect, restoring IFN-γ signaling, increasing MHC-I surface levels, and amplifying CD8⁺ T cell–mediated cytotoxicity. Importantly, PRMT1 inhibition enhances the efficacy of anti-PD-1 treatment in preclinical models, positioning it as a viable target for combination with checkpoint blockade [125]. These complementary therapeutic strategies—activating WHSC1 to reinforce antigen presentation and inhibiting PRMT1 to lift epigenetic repression—offer a dual-pronged approach to enhance tumor immunogenicity. PRMT1 inhibitors, including GSK3368715, are currently being evaluated in early-phase clinical trials [108], while modalities to boost WHSC1 activity or counteract its loss are under preclinical exploration. By reshaping antigen presentation pathways, these PMT-targeted approaches hold promise for converting immune-resistant CRC into immunologically active tumors.

### Disrupt glycosylation enzymes to unmask tumor antigens and sensitize tumors to immunotherapy

Targeting aberrant glycosylation has gained traction as an immunomodulatory approach in CRC, particularly for tumors refractory to immune checkpoint blockade. Several small-molecule inhibitors have been developed to selectively interfere with glycosylation processes that stabilize immune-inhibitory proteins. Among these, NGI-1, a selective inhibitor of the oligosaccharyltransferase complex subunits STT3A and STT3B, has shown robust preclinical efficacy([126]– [127]). Similarly, in head and neck carcinoma, STT3B-mediated glycosylation stabilizes the EREG growth factor, promoting PD-L1 upregulation; treatment with the STT3 inhibitor NGI-1 destabilizes EREG and synergizes with anti-PD-L1 therapy. These findings highlight the feasibility of enzyme-level intervention to destabilize immunosuppressive glycoproteins. Broader reviews and disease models confirm that modulation of PD-L1/PD-1 glycosylation significantly enhances antibody binding and antitumor activity [128]. However, global inhibition of glycosylation may result in systemic toxicity, underscoring the need for target-specific strategies; nanoparticle-based delivery systems and tumor-specific glycosidase conjugates are being explored to confine effects to malignant tissue. Although no glycosylation-targeting agents have yet entered clinical trials for CRC, STT3A/B inhibitors like NGI-1 show promising preclinical activity. The capacity to unmask tumor antigens and sensitize glycoprotein-rich tumors to ICB underscores glycosylation enzymes as emerging immunotherapeutic targets.

### Combine PTM-targeted therapies with immunotherapy to overcome resistance

Given the complexity of immune evasion in CRC, particularly in MSS subtypes, monotherapy with ICIs often achieves limited efficacy. To address this challenge, combinatorial strategies integrating immunotherapy with agents targeting PTM enzymes have gained increasing attention. HDACis, such as entinostat and vorinostat, enhance MHC class I expression and chemokine production, thereby facilitating antigen presentation and T cell infiltration. When administered with PD-1 or CTLA-4 blockade, these agents have been shown to reduce immunosuppressive populations such as Tregs and MDSCs, ultimately improving tumor control in preclinical CRC models. Although early-phase clinical trials have evaluated HDACi–ICI combinations, no trials to date have demonstrated significant efficacy in MSS CRC, underscoring the need for biomarker-guided approaches. Similarly, targeting glycosylation regulators, particularly STT3 and B3GNT3, also sensitizes tumors to immunotherapy: upregulated STT3 promotes PD-L1 N-glycosylation and stabilization in EMT + CRC cells, and inhibition of STT3 (e.g., via KYA1797K) reduces glycosylation and enhances CD8⁺ T cell activity [129]. Furthermore, triplet regimens (e.g., HDACi + anti–PD-1 + chemotherapy, or STT3 inhibitor + anti–PD-L1 + MEK inhibitor) are under investigation to address tumor heterogeneity and redundant escape pathways. Notably, a recent study showed that co-inhibition of JAK and HDAC, in combination with regorafenib, increased acetylation of H3K9, H4K8, and α-tubulin, resulting in enhanced apoptosis, cell cycle arrest, and immune infiltration in CRC, demonstrating the translational relevance of PTM-targeted combinatorial approaches [130].

Beyond histone acetylation and glycosylation, other PTM pathways are also being explored in combination immunotherapy. Targeting the deubiquitinase USP7 with the inhibitor A11 promotes ubiquitin-mediated degradation of PD-L1, thereby enhancing the efficacy of anti–PD-1 immunotherapy. A11 competitively binds PD-L1, inhibiting USP7-mediated deubiquitination, which leads to increased PD-L1 degradation and improved CD8⁺ T cell–mediated tumor cell killing, resulting in synergistic antitumor effects when combined with PD-1 blockade [131]. USP7 interacts with and stabilizes PD-L1 in gastric cancer, and that inhibition of USP7 reduces PD-L1 expression, thereby enhancing T cell-mediated tumor killing while also suppressing tumor cell proliferation via p53 stabilization, suggesting USP7 inhibitors as promising agents to improve PD-1/PD-L1 blockade efficacy [42]. Inhibition of the USP7 by P5091 disrupts a positive feedback loop involving PRDM1-mediated upregulation of FGL1, thereby enhancing CD8⁺ T cell activity and suppressing liver cancer growth; combined USP7 and LAG3 blockade demonstrates superior antitumor efficacy compared to LAG3 inhibition alone [132].

### Advance clinical development while overcoming translational challenges of PTM-based therapies

Targeting agents in CRC is progressing but remains in early stages. Several preclinical and early-phase clinical studies have investigated PTM-targeting agents in combination with immunotherapy for microsatellite-stable (MSS) CRC, where monotherapy responses remain limited. Histone deacetylase inhibitors (HDACis), such as domatinostat, have been explored for their potential to restore MHC expression and enhance T cell infiltration. However, in the EMERGE Phase II trial (NCT03812796) evaluating domatinostat combined with avelumab in MMR-proficient gastrointestinal cancers, no clinical responses were observed in the CRC cohort, and the study did not progress to stage two for this group, underscoring the limited efficacy of this combination in CRC to date [133] (ClinicalTrials.gov).

Inhibitors targeting deubiquitinases such as USP7 and USP22 are currently in early-phase clinical development, supported by preclinical evidence demonstrating enhanced sensitivity to PD-1 blockade when these enzymes are inhibited. Similarly, inhibitors of glycosylation enzymes like STT3, while not yet in clinical trials, have shown robust preclinical activity in destabilizing PD-L1 and restoring antitumor immunity. Despite these advances, multiple translational challenges impede clinical progress. The broad biological functions of PTM enzymes increase the risk of off-target toxicities, necessitating the identification of reliable predictive biomarkers to stratify patients most likely to benefit. Integrative multi-omics approaches combining PTM profiling with transcriptomic and proteomic data hold promises to refine patient selection and therapeutic targeting. Moreover, the pharmacokinetic properties and tumor penetration of many PTM inhibitors remain suboptimal, highlighting the need for improved drug delivery systems such as nanoparticle-based platforms. The development of innovative modalities, including PROTACs and CRISPR-based epigenetic editing, faces additional regulatory and manufacturing complexities that must be addressed. Ultimately, overcoming these hurdles will require coordinated multidisciplinary collaboration among academic researchers, pharmaceutical developers, and regulatory bodies to translate PTM-targeted therapies into effective immunotherapeutic options for colorectal cancer patients.

## Concluding remarks and future perspectives

PTMs have emerged as pivotal regulators of CRC biology, intricately controlling immune evasion, metabolic reprogramming, and tumor progression. By dynamically modulating key proteins involved in antigen presentation, immune checkpoint stability, signaling cascades, and metabolic enzymes, PTMs shape the tumor microenvironment and influence therapeutic responses [143]. This multifaceted regulatory layer adds complexity to CRC pathogenesis but also offers a rich reservoir of potential biomarkers and therapeutic targets.

Recent advances in proteomic technologies, single-cell analyses, and spatial profiling have significantly enhanced our understanding of the diverse and context-dependent roles of PTMs in CRC. Integration of PTM data with genomic and transcriptomic information through multi-omics approaches is refining patient stratification and enabling more precise prediction of immunotherapy responses. These technological innovations not only illuminate fundamental mechanisms of tumor-immune interactions but also guide the rational design of next-generation therapeutic strategies.

Clinically, targeting PTM enzymes such as histone deacetylases, ubiquitin ligases, deubiquitinases, kinases, phosphatases, and glycosyltransferases holds considerable promise to overcome resistance to current immunotherapies and conventional treatments. Early-phase clinical trials combining PTM inhibitors with immune checkpoint blockade have demonstrated encouraging immunomodulatory effects, particularly in microsatellite stable CRC subtypes that traditionally show poor responsiveness. However, significant challenges remain before widespread clinical adoption. The pleiotropic functions and ubiquitous nature of many PTM enzymes raise concerns about systemic toxicity and off-target effects, necessitating the development of highly selective inhibitors and targeted delivery methods.

Tumor heterogeneity and temporal dynamics of PTMs complicate biomarker identification and therapeutic timing, underscoring the need for longitudinal and spatially resolved molecular monitoring. Moreover, adaptive resistance mechanisms frequently emerge through compensatory signaling pathways or epigenetic remodeling, indicating that single-agent PTM inhibition may be insufficient. Rational combination therapies, integrating PTM-targeted agents with immune checkpoint inhibitors, chemotherapy, targeted kinase inhibitors, and emerging modalities such as PROTACs or epigenetic editors, are likely essential to achieve durable clinical benefit.

Looking forward, the successful translation of PTM-based therapies in CRC will depend on interdisciplinary collaboration bridging basic research, technology development, and clinical oncology. Advances in artificial intelligence and machine learning applied to integrated multi-omics datasets will accelerate biomarker discovery and patient selection, while innovative drug design and delivery platforms will improve therapeutic specificity and safety. As our mechanistic understanding of PTM regulation deepens, novel vulnerabilities within tumor and immune compartments will be uncovered, paving the way for personalized immuno-oncology strategies. In conclusion, PTMs represent a promising frontier in colorectal cancer research and therapy. Harnessing their regulatory complexity through cutting-edge technologies and translational efforts offers a compelling path to enhance immunotherapy efficacy, overcome therapeutic resistance, and ultimately improve patient outcomes.

## Data Availability

The clinical trial data comes from the website: www.ClinicalTrials.gov.
